# Supplementary material for: Identification of Freezing-Responsive microRNAs and Their Targets in Chinese Jujube by Small RNA and Degradome Sequencing
Source: Int J Mol Sci. 2026 Sep 4;27(17):7912. doi: 10.3390/ijms27177912 (PMC13566890; doi:10.3390/ijms27177912)
Supplement: Supplementary file 1 [file ijms-27-07912-s001.zip › Supplementary Material S2/novel_miR_230.pdf]

Provisional ID	:	novel_miR_230
Score total	:	1427.4
Score for star read(s)	:	3.9
Score for read counts	:	1425.6
Score for mfe	:	0.1
Score for randfold	:	-2.2
Score for cons. seed	:	
Total read count	:	2808
Mature read count	:	2796
Loop read count	:	0
Star read count	:	12

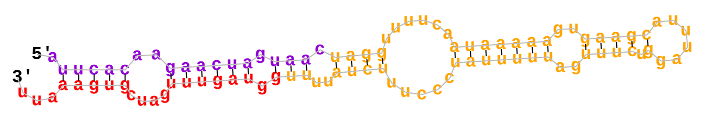

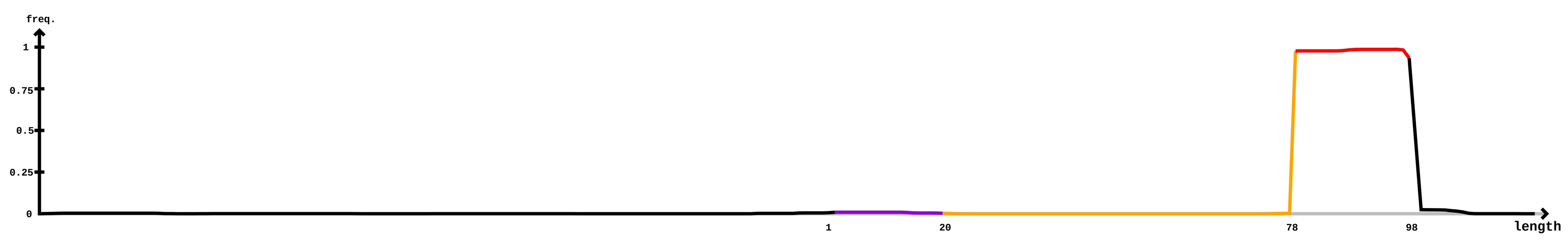

Star

Mature

```
5'- guaaaguagcuccugggugggaaacuacuccuuugauggguaucgcaauggcccuuuuugcauuauuccuaucuauuauuuuggagauuuauacuucuuccauuuuacuggacaaauuuggaaugaauuagauucacaagaacuaguaacuagguuuucaauaaaaagugaagcauuuaggucuuugauuuuuaucccuuucuauuuugguaguuugaucgugaaauuucuuuguuuuguaguuuugaa -3' obs
    guaaaguagcuccugggugggaaacuacuccuuugauggguaucgcaauggcccuuuuugcauuauuccuaucuauuauuuuggagauuuauacuucuuccauuuuacuggacaaauuuggaaugaauuagauucacaagaacuaguaacuagguuuucaauaaaaagugaagcauuuaggucuuugauuuuuaucccuuucuauuuugguaguuugaucgugaaauuucuuuguuuuguaguuuugaa     exp
    ...(((((.((((((((((((((..(((.((......)))))..((((.........))))....))))))))))).......))))....)))))..((((.....))))((((.(((.((.((...((((((((..((((((.(((.((((......(((((((..(((((......).))))..))))))).....))))..))).))))))....)))))...)))...)).)).))).))))..
```

read	reads	mm	sample
guaaaguagcuccugggu	2	0	LT-3
guaaaguagcuccugggug	1	0	JCK-1
guaaaguagcuccugggug	1	0	JCK-2
.uaaaguagcuccugggug	3	0	JCK-2
..aaaguagcuccugggugg	2	0	LT-1
uacuccuuugauggguaucgcaauggc	1	0	LT-2
cuccuuugauggguaucgcaaugg	1	0	JT-3
auuccuaucuauuauuuuggagau	1	0	JCK-2
uggaaugaauuagauucacaagaacu	2	0	LT-3
uggaaugaauuagauucacaagaacu	1	0	LT-2
uggaaugaauuagauucacaagaacua	2	0	LT-2
uggaaugaauuagauucacaagaacuagua	1	0	LT-1
aauuagauucacaagaac	3	0	LCK-3
aauuagauucacaagaacu	1	0	LT-2
aauuagauucacaagaacu	1	0	JCK-2
aauuagauucacaagaacu	1	0	JCK-3
aaNuagauucacaagaacu	1	1	LT-2
auuagauucacaagaacu	1	0	JCK-2
gauucacaagaacuaguaa	2	0	JT-2
gauucacaagaacuaguaa	2	0	JT-3
auucacaagaacuaguaac	4	0	JCK-1
auucacaagaacuaguaac	1	0	LT-3
auucacaagaacuaguaacu	2	0	LT-3
auucacaagaacuaguaacua	1	0	LT-3
uuuugguaguuugaucgugaaauu	1	0	JCK-1
uuuugguaguuNgaucgugaaauu	1	1	JCK-1
uuugguaguuugaucgugGaauu	2	1	LCK-2
uugguaguuugaucgugGaauu	1	1	LCK-2
uugguaguuugaucgugGaauu	1	1	LT-1
ugguaguuugaucgugGaauu	1	1	JCK-3
gguaguuugaucgugGaa	3	1	LT-1
gguaguuugaucgugGaa	8	1	LCK-2
gguaguuugaucgugGaau	1	1	JCK-1

```
                                                                                                                                   Star                                                                         Mature
guaaaguagcuccugggugggaaacuacuccuuugauggguaucgcaauggcccuuuuugcauuauuccuaucuauuauuuuggagauuuauacuucuuccauuuuacuggacaaauuuggaaugaauuagauucacaagaacuaguaacuagguuuucaauaaaaagugaagcauuuaggucuuugauuuuuaucccuuucuauuuugguaguuugaucgugaaauuucuuuguuuuguaguuugaa
................................................................................................................................................................................................................gguaguuugaucgugaaau.....................   1      0   LT-2
................................................................................................................................................................................................................gguaguuugaucgugGaau.....................   46     1   LCK-2
................................................................................................................................................................................................................gguaguuugaucgugGaau.....................   4      1   LCK-3
................................................................................................................................................................................................................gguaguuugaucgugGaau.....................   14     1   JT-3
................................................................................................................................................................................................................gguaguuugaucgugUaau.....................   3      1   JT-2
................................................................................................................................................................................................................gguaguuugaucgugGaau.....................   7      1   JT-2
................................................................................................................................................................................................................gguaguuugaucgugGaau.....................   18     1   LT-1
................................................................................................................................................................................................................gguaguuugaucgugUaau.....................   2      1   JT-3
................................................................................................................................................................................................................gguaguuugaucgugGaau.....................   23     1   JT-1
................................................................................................................................................................................................................gguaguuugaucgugGaau.....................   6      1   JCK-2
................................................................................................................................................................................................................gguaguuugaucgugGaau.....................   8      1   LT-3
................................................................................................................................................................................................................gguaguuugaucgugUaau.....................   7      1   JCK-2
................................................................................................................................................................................................................gguaguuugaucgugaaauu....................   13     0   JCK-1
................................................................................................................................................................................................................gguaguuugaucgugGaauu....................   45     1   JT-3
................................................................................................................................................................................................................gguaguuugaucgugUaauu....................   16     1   JT-3
................................................................................................................................................................................................................gguaguuugaucgugGaauu....................   517    1   LT-1
................................................................................................................................................................................................................gguaguuugaucgugGaauu....................   7      1   LCK-1
................................................................................................................................................................................................................gguaguuugaucgugaaauu....................   8      0   LCK-3
................................................................................................................................................................................................................gguaguuGgaucgugaaauu....................   1      1   LT-2
................................................................................................................................................................................................................gguaguuugaucgugUaauu....................   6      1   JCK-2
................................................................................................................................................................................................................gguaguuugaucgugaaauu....................   4      0   LT-2
................................................................................................................................................................................................................gguaguuugaucgugGaauu....................   199    1   LT-3
................................................................................................................................................................................................................gguaguuugaucgugaaauu....................   1      0   LCK-2
................................................................................................................................................................................................................gguaguuugaucgugUaauu....................   5      1   JCK-1
................................................................................................................................................................................................................gguaguuugaucgugaaauu....................   18     0   JCK-2
................................................................................................................................................................................................................gguaguuugaucgugaaauu....................   9      0   JCK-3
................................................................................................................................................................................................................gguaguuAgaucgugaaauu....................   1      1   LT-3
................................................................................................................................................................................................................gguaguuAgaucgugaaauu....................   1      1   JCK-1
................................................................................................................................................................................................................gguCguuugaucgugaaauu....................   1      1   JCK-1
................................................................................................................................................................................................................gguaguuugaucgugUaauu....................   10     1   JT-2
................................................................................................................................................................................................................gguaguuuAaucgugaaauu....................   1      1   LT-1
................................................................................................................................................................................................................gguaguuugaucgugUaauu....................   1      1   LT-1
................................................................................................................................................................................................................gguaguuugaucgugGaauu....................   41     1   JT-2
................................................................................................................................................................................................................gguaguuugaucgugGaauu....................   6      1   LT-2
................................................................................................................................................................................................................gguaguuugaucgugGaauu....................   1304   1   LCK-2
................................................................................................................................................................................................................gguaguuAgaucgugaaauu....................   1      1   JT-3
................................................................................................................................................................................................................gguaguuAgaucgugaaauu....................   1      1   JCK-3
................................................................................................................................................................................................................gguaguuugaucgugGaauu....................   26     1   JCK-1
................................................................................................................................................................................................................gguaguuugaucgugaaauu....................   3      0   LT-1
................................................................................................................................................................................................................gguaguuugaucgugGaauu....................   55     1   JCK-2
................................................................................................................................................................................................................gguaguuugaucgugGaauu....................   8      1   JCK-3
................................................................................................................................................................................................................gguaguuugaucgugGaauu....................   120    1   JT-1
................................................................................................................................................................................................................gguaguuugaucgugGaauu....................   203    1   LCK-3
................................................................................................................................................................................................................gguaguuugaucgugGaauuu...................   1      1   LCK-3
................................................................................................................................................................................................................gguaguuugaucgugaaauuu...................   1      0   LT-2
................................................................................................................................................................................................................gguaguuugaucgugaaauuu...................   1      0   JCK-2
................................................................................................................................................................................................................gguaguuugaucgugGaauuu...................   1      1   LT-3
................................................................................................................................................................................................................gguaguuugaucgugaaauuuc..................   1      0   JCK-2
................................................................................................................................................................................................................gguaguuugaucgugaaauuucu.................   1      0   LT-1
................................................................................................................................................................................................................gguaguuugaucgugaaauuucuu................   1      0   LT-2
................................................................................................................................................................................................................gguaguuugaucgugaaauuucuuu...............   1      0   LT-3
................................................................................................................................................................................................................gguaguuugaucgugGaauuucuuu...............   1      1   JCK-2
................................................................................................................................................................................................................gguaguuugaucgugGaauuucuuug..............   2      1   LCK-2
................................................................................................................................................................................................................Aguaguuugaucgugaaauuucuuug..............   1      1   LT-1
................................................................................................................................................................................................................gguaguuugaucgugaaauuucuuug..............   5      0   JCK-3
................................................................................................................................................................................................................gguaguuugaucgugaaauuucuuug..............   1      0   LT-1
................................................................................................................................................................................................................gguaguuugaucgugaaauuucuuug..............   1      0   JCK-2
................................................................................................................................................................................................................gguaguuAgaucgugaaauuucuuugu.............   2      1   JCK-2
................................................................................................................................................................................................................ggGaguuugaucgugaaauuucuuugu.............   1      1   JCK-1
................................................................................................................................................................................................................gguaguuugaucgugaaauuucuuugu.............   1      0   JCK-2
................................................................................................................................................................................................................gguaguuugaucgugaaauuucuuugu.............   2      0   JCK-1
................................................................................................................................................................................................................gguaguuugaucgugaaauuucuuugu.............   2      0   LT-2
................................................................................................................................................................................................................gguaguuugaucgugaaauuucuuuguu............   1      0   LT-1
................................................................................................................................................................................................................gguaguuuAaucgugaaauuucuuuguu............   1      1   LT-3
................................................................................................................................................................................................................gguaguuugaucgugGaauuucuuuguu............   1      1   LCK-2
................................................................................................................................................................................................................gguaguuugaucgugaaauuucuuuguu............   1      0   JCK-3
................................................................................................................................................................................................................gguaguuugaucgugGaauuucuuuguu............   1      1   JCK-3
................................................................................................................................................................................................................gguaguuAgaucgugaaauuucuuuguuu...........   1      1   JCK-2
................................................................................................................................................................................................................gguaguuugaucgugaaauuucuuuguuu...........   1      0   JCK-1
................................................................................................................................................................................................................gguaguuAgaucgugaaauuucuuuguuu...........   1      1   LT-3
```

```
                                                                                                                                                                   Star                                                                                 Mature
guaaaguagcuccugggugggaaacuacuccuuugauggguaucgcaauggcccuuuuugcauuauuccuaucuauuauuuuggagauuuauacuucuuccauuuuacuggacaaauuuggaaugaauuagauucacaagaacuaguaacuagguuuucaauaaaaagugaagcauuuaggucuuugauuuuuaucccuuucuauuuugguaguuugaucgugaaauuucuuuguuuuguaguuugaa
................................................................................................................................................................................................................gguaguuAgaucgugaaauucuuuguuu............   1   1   LT-1
................................................................................................................................................................................................................gguaguuugaucgugaaauucuuuguuu............   3   0   JCK-2
................................................................................................................................................................................................................gguaguuugaucgugaaauucuuuguuu............   1   0   JCK-3
................................................................................................................................................................................................................gguaguuugaucgugGaauucuuuguuu............   1   1   LT-1
................................................................................................................................................................................................................gguaguuCgaucgugaaauucuuuguuuu...........   1   1   LT-2
................................................................................................................................................................................................................gguaguuCgaucgugaaauucuuuguuuu...........   1   1   JCK-1
................................................................................................................................................................................................................gguaguuCgaucgugaaauucuuuguuuu...........   1   1   JCK-3
................................................................................................................................................................................................................gguaguuCgaucgugaaauucuuuguuuu...........   3   1   JCK-2
.................................................................................................................................................................................................................guaguuugaucgugGaauu....................   1   1   LT-1
.......................................................................................................................................................................................................................gaucgugaaauucuuug................   1   0   LT-3
.......................................................................................................................................................................................................................gaucgugaaauucuuuguuu.............   1   0   JCK-2
.......................................................................................................................................................................................................................gaucgugaaauucuuuguuu.............   4   0   JCK-3
........................................................................................................................................................................................................................aucgugaaauucuuugu...............   2   0   LT-2
........................................................................................................................................................................................................................aucgugaaauucuuuguu..............   3   0   LT-3
........................................................................................................................................................................................................................aucgugaaauucuuuguuu.............   3   0   JCK-1
........................................................................................................................................................................................................................aucgugaaauucuuuguuu.............   2   0   LT-1
........................................................................................................................................................................................................................aucgugaaauucuuuguuu.............   1   0   LT-2
........................................................................................................................................................................................................................aucgugaaauucuuuguuu.............   1   0   LT-3
.........................................................................................................................................................................................................................ucgugaaauucuuuguu..............   5   0   LCK-3
.........................................................................................................................................................................................................................ucgugaaauucuuuguu..............   1   0   JCK-2
..........................................................................................................................................................................................................................cgugaaauucuuuguuu.............   2   0   LT-2
...............................................................................................................................................................................................................................auuucuuuguuuCuguaguuu....   1   1   LT-3
```
